# Kierkegaard and Anxiety: Bridging Philosophy, Psychotherapy, and Art

**DOI:** 10.1192/j.eurpsy.2025.1936

**Published:** 2025-08-26

**Authors:** F. Cunha, I. Santos, R. Cabral, P. Pires, C. Cunha, S. Borges

**Affiliations:** 1 ULS Viseu Dão-Lafões, Viseu, Portugal

## Abstract

**Introduction:**

Søren Kierkegaard’s philosophy, particularly his exploration of anxiety, serves as a cornerstone in existential thought. His concepts of “authenticity,” “choice,” and the confrontation with the self underlie much of the contemporary understanding of anxiety as both a psychological and philosophical experience. Kierkegaard’s works, especially *Either/Or*, offer a profound analysis of human freedom, responsibility, and the resulting anxiety. The objective of this analysis is to bridge the philosophical with the practical and artistic, providing a multidimensional understanding of anxiety.

**Objectives:**

This study seeks to explore Kierkegaard’s conceptualization of anxiety, its application in psychotherapy, and its resonance in contemporary culture. Specifically, it investigates how these ideas can aid individuals in confronting existential anxiety in therapeutic settings.

**Methods:**

A philosophical and qualitative approach is used, analyzing Kierkegaard’s *Either/Or.* The study incorporates interpretations from existential thinkers, psychotherapists and artists. Theoretical analysis is coupled with psychological insights, exploring anxiety as a pathway to self-awareness.

**Results:**

Kierkegaard presents anxiety as inherent to human freedom and choice. His stages of life—the aesthetic, ethical, and religious—represent different ways of engaging with anxiety. In the aesthetic stage, individuals pursue pleasure but encounter despair when they confront their limitations. The ethical stage offers structure but introduces existential guilt. The religious stage, requiring a leap of faith, is seen as the highest form of existence, where anxiety leads to transcendence. Kierkegaard’s ideas are reflected in existential psychotherapy, where anxiety is viewed not only as a symptom but as a catalyst for personal growth. Elliott Smith’s *Either/Or* album mirrors these themes, portraying modern struggles with freedom, despair, and self-doubt. Songs like “Between the Bars” and “Ballad of Big Nothing” articulate the tension between aesthetic escape and ethical responsibility, offering a visceral interpretation of Kierkegaard’s philosophical concerns.

**Conclusions:**

Kierkegaard’s exploration of anxiety offers profound insights into human existence, emphasizing the need to confront, rather than avoid, existential dilemmas. His framework of life stages provides a guide for understanding the progression toward authenticity, where anxiety is a key driver. The study shows that Kierkegaard’s ideas remain relevant in psychotherapy, helping individuals navigate anxiety to achieve self-realization. Ultimately, this research affirms that embracing anxiety is crucial for personal freedom, growth, and authenticity, aligning with Kierkegaard’s vision of a life well-lived.

**Disclosure of Interest:**

None Declared

